# A Highly Polymorphic Panel Consisting of Microhaplotypes and Compound Markers with the NGS and Its Forensic Efficiency Evaluations in Chinese Two Groups

**DOI:** 10.3390/genes11091027

**Published:** 2020-09-01

**Authors:** Xiaoye Jin, Xingru Zhang, Chunmei Shen, Yanfang Liu, Wei Cui, Chong Chen, Yuxin Guo, Bofeng Zhu

**Affiliations:** 1 Key Laboratory of Shaanxi Province for Craniofacial Precision Medicine Research, College of Stomatology, Xi’an Jiaotong University, Xi’an 710004, China; jinxy0901@stu.xjtu.edu.cn (X.J.); olliecheung@stu.xjtu.edu.cn (X.Z.); cuiwei3702@stu.xjtu.edu.cn (W.C.); cc18883368974@stu.xjtu.edu.cn (C.C.); guoyuxin_004@stu.xjtu.edu.cn (Y.G.); 2 College of Forensic Medicine, Xi’an Jiaotong University Health Science Center, Xi’an 710061, China; 3 Clinical Research Center of Shaanxi Province for Dental and Maxillofacial Diseases, College of Stomatology, Xi’an Jiaotong University, Xi’an 710004, China; 4 Institute of Brain and Behavioral Sciences, College of Life Sciences, Shaanxi Normal University, Xi’an 710062, China; cmshen2004@snnu.edu.cn; 5 Multi-Omics Innovative Research Center of Forensic Identification, Department of Forensic Genetics, School of Forensic Medicine, Southern Medical University, Guangzhou 510515, China; liuyanfang92@i.smu.edu.cn

**Keywords:** forensic research, microhaplotype, compound marker, Kazak, Mongolian

## Abstract

Novel genetic markers like microhaplotypes and compound markers show promising potential in forensic research. Based on previously reported single nucleotide polymorphism (SNP) and insertion/deletion (InDel) polymorphism loci, 29 genetic markers including 22 microhaplotypes and seven compound markers were identified. Genetic distributions of the 29 loci in five continental populations, Kazak and Mongolian groups in China were investigated. We found that the expected heterozygosity values of these 29 loci were >0.4 in these populations, indicating these loci were relatively high polymorphisms. Population genetic analyses of five continental populations showed that five loci displayed relatively high genetic variations among these continental populations and could be useful markers for ancestry analysis. In summary, the 29 loci displayed relatively high genetic diversities in continental populations and Chinese two groups and could be informative loci for forensic research.

## 1. Introduction

In forensic research, human identification and paternity testing are two important research items. Since short tandem repeats (STRs) are highly polymorphic and widely distributed in the human genome, they are universally employed in forensic practice [1,2]. However, there are some deficiencies of STRs in the application. For example, their relatively longer amplicon lengths make the detection difficult in degraded DNA samples, which may lead to the loss of some alleles with long amplicon lengths [3], and the high mutation rate of STRs may bring about difficulty in paternity analyses [4]. Compared to STRs, single nucleotide polymorphisms (SNPs) and insertion/deletion (InDel) polymorphisms possess some favorable characteristics like a relatively low mutation rate and small amplicon size, and they have been paid considerable attention by forensic geneticists [5,6,7,8,9]. Even so, SNPs and InDels commonly demonstrate di-allelic variations, which lead to low polymorphisms. Therefore, more SNPs and InDels need to be identified to meet the forensic efficiency of commonly used STRs.

Forensic geneticists recently explored the application values of some novel genetic markers in forensic practice. Liu et al. proposed a novel compound marker that was a combination of one InDel and one SNP in a genomic region; they evaluated the power of the novel genetic marker to detect the DNA mixture, and their results revealed that the novel marker was able to disentangle the unbalanced mixture [10]. Microhaplotypes, defined by two or more closely linked SNPs, refer to short DNA segments (<300 nucleotides) [11]. Allele amplicons of microhaplotypes are commonly shorter than those of STRs, which suggest they can be utilized in degraded samples because alleles with short amplicons could be successfully amplified. Moreover, there are no polymerase slippages in polymerase chain reaction (PCR) of microhaplotype, so no stutter peaks are observed in microhaplotype analyses [12]. More importantly, microhaplotypes have multiple allele variations, which further improve their genetic diversities compared with a single SNP or InDel locus. Considerable research on the forensic effectiveness of microhaplotypes has been conducted in recent years. Turchi et al. selected 89 microhaplotypes, evaluated their genetic distributions in the Italian population using the next generation sequencing (NGS) and found that these loci showed great potential in forensic individual identification [13]. Chen et al. chose some microhaplotypes with high effective numbers of alleles (Ae) for mixture deconvolution based on NGS and found that these loci could distinguish between minor and the major contributors [14]. Pang et al. constructed a multiplex system of 124 microhaplotypes based on NGS and compared the forensic efficiency of these loci with commonly used STRs; their results demonstrated that 20 microhaplotypes with top Ae values possessed similar power to differentiate unrelated individuals in comparison with 20 STRs [15]. Cheung et al. compared performances of microhaplotypes and SNPs for ancestry analyses of different continental populations and concluded that microhaplotypes showed the highest performances for ancestry analyses of five continental populations [16]. Zhu et al. explored the effectiveness of microhaplotypes in kinship analysis and found that 11 novel selected microhaplotypes possessed high application values [17]. In summary, microhaplotypes and compound markers show great potential in forensic research, but more loci must be identified for forensic application.

In the present study, 29 novel microhaplotypes and compound markers (InDel-SNP) were selected from the dbSNP database (https://www.ncbi.nlm.nih.gov/snp) based on previously reported SNPs [18,19,20,21,22,23] and InDels [24,25]. Genetic distributions and forensic efficiencies of these loci in different continental populations were evaluated, and then population genetic analyses of these continental populations were performed based on the selected loci. Next, a multiplex amplification system consisting of these 29 loci was developed using NGS, and 112 Kazak and 106 Mongolian individuals in China were detected. Finally, we assessed the forensic application values of these 29 loci in both studied groups.

## 2. Materials and Methods

### 2.1. Selection of Novel Microhaplotypes and Compound Markers

Based on previously reported SNP [18,19,20,21,22,23] and InDel loci [24,25], we selected the loci with neighboring regions (<200 bp) that had polymorphic SNP or InDel loci (minor allele frequency >0.01). These loci selected initially were further screened using the following criteria: (1) located in intronic regions, (2) different allelic frequencies of SNPs/InDels in the same region, (3) conform to Hardy–Weinberg equilibrium (HWE) in East Asian population [26], and (4) the polymorphism information content (PIC) value of each locus is larger than 0.5 in East Asian population. Finally, we identified the 29 novel genetic markers including the 22 microhaplotypes and seven compound markers that were used to construct the multiplex amplification system based on NGS platform.

### 2.2. Sample Preparation and DNA Extraction

Blood samples were collected from 112 Kazaks and 106 Mongolians living in northwest China after obtaining their written informed consent. There were no blood kinships among analyzed participants according to their self-descriptions. Genomic DNA was extracted using Magbead Blood Spots DNA kit (CWBIO, Beijing, China). A NanoDrop 2000 instrument (Thermo Fisher Scientific, Waltham, MA, USA) was utilized to determine the concentration of each DNA sample. PCR primers for each region were designed on Primer 6.0 software. The primer sequences used in this study are given in Supplementary Table S1. The study fully complied with the human and ethical research principles of Xi’an Jiaotong University Health Science Center, China (XJTULAC201, 2019–1039).

### 2.3. Reference Populations

Five continental populations (including African, American, East Asian, European and South Asian) were used as reference populations for the initial evaluations of genetic distributions of selected SNPs/InDels. Genetic genotypes of all the selected SNPs/InDels in these continental populations were obtained from 1000 Genome Project Phase 3 [26].

### 2.4. Libraries Construction and Sequencing Using the NGS

The sequencing library of each sample was prepared according to the following instructions. The total PCR system was 25 μL, consisting of 12.5 μL 2× Platinum multiplex PCR master mix, 3 μL GC enhancer, 2.5 μL primer mix (2 μM), 10 ng genomic DNA and ddH2O (up to 25 μL). We performed thermal cycling with the following conditions: denaturation for 2 min at 95 °C; 35 cycles of 30 s at 95 °C, 90 s at 60 °C and 30 s at 72 °C; extension was performed for 5 min at 72 °C. After PCR, we used 2% agarose gel electrophoresis to segregate DNA segments, and magnetic beads were used to purify DNA samples using CMPure MagBead DNA Purification kit (CWBIO, Beijing, China). Next, we conducted the second round amplification based on KAPA HiFi HotStart ReadyMixPCR kit (Kapa Biosystems, Boston, MA, USA). The reaction reagents were 12.5 μL 2× KAPAHIFI mix, 2.5 μL Barcode (50 μM), 2.5 μL PE 1.0 (50 μM), 5 μL purified PCR product and 2.5 μL ddH_2_O. PCR was conducted on the GeneAmp PCR System 9700 based on the following parameters: 98 °C for 2 min; 8 cycles of 98 °C for 20 s, 65 °C for 30 s, 72 °C for 20 s; 72 °C for 5 min and hold at 4 °C. Then constructed DNA libraries were separated using 2% agarose gel electrophoresis. We further purified DNA libraries using CMPure MagBead DNA Purification kit (CWBIO, Beijing, China). The Qubit dsDNA HS Assay kit (Thermo Fisher Scientific, Waltham, MA, USA) was employed to quantify the concentration of each library.

We denatured and diluted libraries using the standard normalization method. The final concentration of the library pool was 1.8 pM. Moreover, 1% PhiX control was used as the quality control and added to the library pool. The detailed instructions were referenced in the NextSeq System Denature and Dilute Libraries Guide (https://support.illumina.com.cn/sequencing/sequencing_instruments/nextseq-500/documentation.html?langsel=/cn/).

The NextSeq 500 High Output kit v2.5 (Illumina, Inc., San Diego, CA, USA) was used to conduct paired-end sequencing (150×) of each sample on the Illumina NextSeq 500 platform (Illumina, Inc., San Diego, CA, USA). The Local Run Manager was used as the run mode to perform sequencing reactions. The number of cycles was 300. We removed reads with self-ligation primer, low quality, multiple N and very short sequences using Cutadapt (http://code.google.com/p/cutadapt/). We compared clean data with the reference genome (h19) using the BWA (http://bio-bwa.sourceforge.net/). We annotated all detected SNP and InDel loci with GATK (https://software.broadinstitute.org/gatk/) and VarScan software packages [27].

### 2.5. Statistical Analyses

We used PHASE software version 2.1 [28] to conduct haplotype reconstruction of each region in different intercontinental populations and the studied Kazak and Mongolian groups. The distribution information of the selected 29 loci on different chromosomes was plotted using the RCircos package [29] in *R* software v3.3 [30]. Expected heterozygosity (He), discrimination power (DP), probability of exclusion (PE) and PIC values of 29 loci in different intercontinental populations were calculated with STRAF online program v1.0.5 [31]; Ae was calculated based on a previous report [32]. Boxplots of He and PIC values and the Ae heatmap of 29 loci in different continental populations were drawn with the ggplot2 [33] and pheatmap packages [34] in *R* software, respectively. Principal component analysis (PCA) of different continental populations was built using STRAF online program based on estimated haplotypic data. We conducted genetic structure analyses from *K* = 2 to *K* = 5 with five independent replicates using STRUCTURE software v2.3.4 [35]. The detailed parameters in STRUCTURE software were 10,000 burn-ins and 10,000 MCMC replications with the admixture, allelic frequency correlated model. We determined the best *K* value with the STRUCTURE HARVESTER online program (http://taylor0.biology.ucla.edu/structureHarvester/). We processed the data of the STRUCTURE replicated run to reduce stochastic effects with CLUMPP software v1.1 [36]. Then the graphic display of CLUMPP outputs was performed with the CLUMPAK online program [37]. We calculated informativeness (*In*) value of each locus in five intercontinental populations with the INFOCALC program [38]. Finally, we estimated haplotypic frequencies, PIC, DP, PE, He, observed heterozygosity (Ho), match probability (MP), *p*-values for HWE and linkage disequilibrium (LD) tests of these 29 loci in Kazak and Mongolian groups using STRAF online program. The Ae values of 29 loci in Kazak and Mongolian groups were calculated based on the description mentioned above. The allele coverage ratio (ACR) of each SNP/InDel was estimated according to a published description [39].

## 3. Results

### 3.1. General Information of the 29 Microhaplotypes and Compound Markers

**Figure 1:**
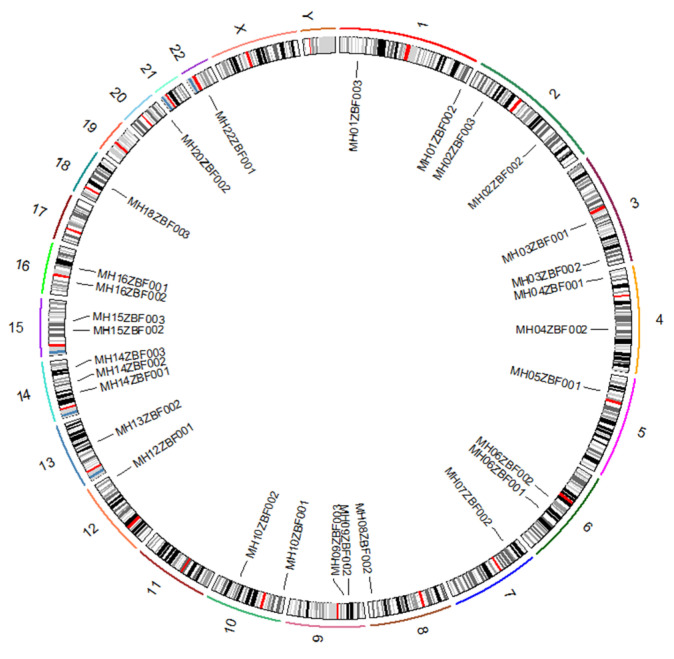
Physical positions of the 29 loci in different chromosomes.

In the present study, 29 microhaplotypes and compound markers were identified from previously reported SNP [18,19,20,21,22,23] and InDel loci [24,25]. We named each locus based on the nomenclature criteria proposed by Kidd et al. [40]. These 29 loci consisted of 69 SNP/InDel loci, and their chromosomal location information is presented in Supplementary Table S2. The results revealed that these 29 loci included 22 microhaplotypes (one InDel-InDel and 21 SNP-SNP markers) and seven compound markers (InDel-SNP); the numbers of SNP/InDel in each locus ranged from 2 to 5. The distribution patterns of these 29 loci in different chromosomes are displayed in Figure 1. The results indicated that they were located in 18 different autosomes.

### 3.2. Genetic Diversities and Forensic Efficiencies of 29 Loci in Five Continental Populations

**Figure 2:**
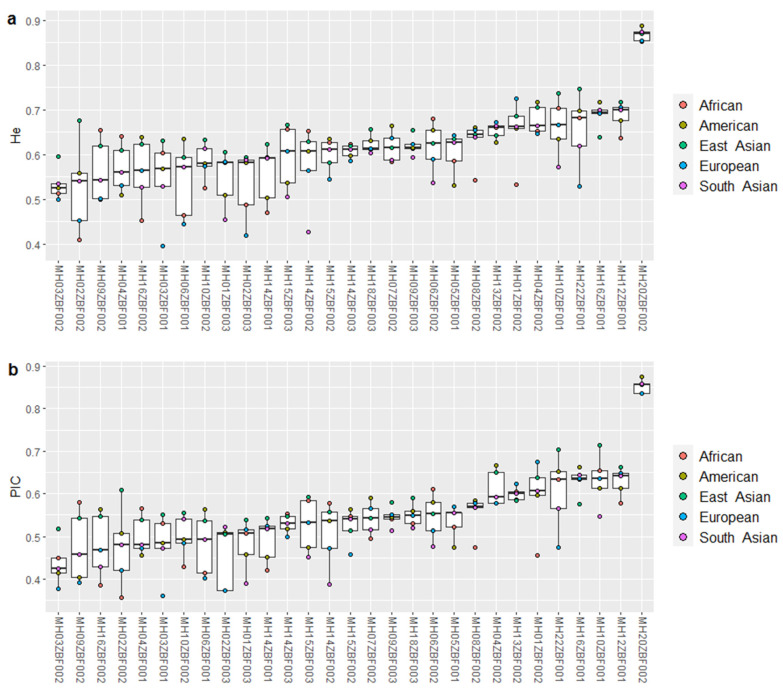
Boxplots of (**a**) expected heterozygosity and (**b**) polymorphism information content of the 29 loci in five continental populations.

Based on the population genetic data reported in 1000 Genomes Phase 3 [26], we assessed genetic distributions of the selected 29 loci in five continental populations. First, we displayed the He values (Figure 2a) and found that they were >0.4 for all loci in these populations, with the highest value for MH20ZBF002 (>0.85) and the lowest for MH03ZBF002. Next, we calculated the PIC values of the 29 loci in these populations (Figure 2b). Similar to the He distribution patterns in these populations, MH20ZBF002 had the highest PIC value, while MH03ZBF002 was relatively low. Even so, the He and PIC values of the 29 loci were greater than 0.5 in East Asian population, implying that they had relatively high genetic diversities in East Asian population. We also analyzed Ae values of the 29 loci in these five continental populations (Supplementary Figure S1). Nineteen loci had relatively high Ae values (>2), with the highest value for MH20ZBF002 (>6), indicating that the locus showed more even allele distributions in these populations and could be utilized for mixture sample analysis.

We also calculated the cumulative discrimination power (CDP), cumulative match probability (CMP) and cumulative probability of exclusion (CPE) values of the selected 29 loci in these five continental populations (Table 1). The CDP values of the 29 loci ranged from 0.99999999999999999982977 in the European population to 0.99999999999999999999968073 in the East Asian population. The CMP values of these loci ranged from 3.1928E-22 in the East Asian population to 1.7023E-19 in the European population. The CPE values distributed from 0.999954 in the European population to 0.999998 in the East Asian population.

### 3.3. Genetic Divergences and Population Structure Evaluations of Different Continental Populations

**Figure 3:**
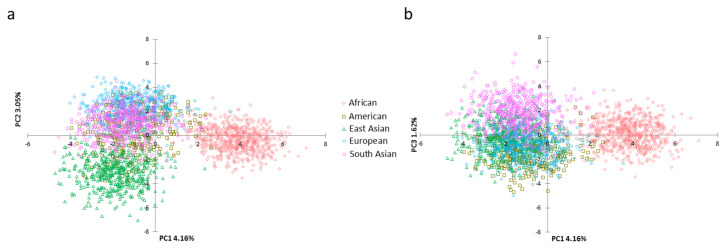
Principal component analysis of five continental populations at (**a**) PC1 and PC2 and (**b**) PC1 and PC3 based on the same 29 loci.

**Figure 4:**
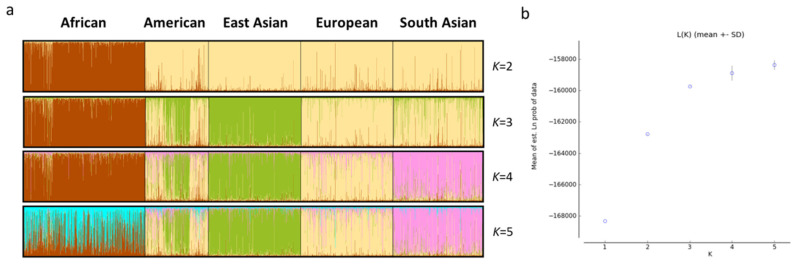
Genetic structure analyses of five continental populations at (**a**) *K* = 2–5 and (**b**) L(*K*) value of each *K* based on the same 29 loci.

Based on haplotypic frequencies of the 29 loci, we conducted PCA of five continental populations (Figure 3). We found that PC1 on the horizontal axis could distinguish African individuals from the other individuals, PC2 (Figure 3a) on the vertical axis could differentiate East Asian individuals from other individuals and PC3 (Figure 3b) could differentiate some South Asian individuals from other individuals. Next, we further explored the genetic structures of these continental populations (Figure 4a). Ancestral components (brown color) in the African population could be discerned at *K* = 2 in comparison with other continental populations mainly showing yellow ancestral components. When *K* increased to 3, the East Asian population could be separated from other populations. As *K* reached 4, African, East Asian, European and South Asian populations displayed different ancestral components: African for brown, East Asian for green, European for yellow, South Asian for pink; American population showed admixed ancestral proportions. The STRUCTURE HARVESTER results are shown in Figure 4b. Similar L(*K*) values could be discerned at *K* = 3–5, indicating that *K* = 3 was the most suitable for the data in this study. The population genetic analyses mentioned above suggested that these 29 loci showed different genetic distributions in these continental populations, which might be useful for differentiating these continental populations. We also estimated the *In* values of the 29 loci among five continental populations (Supplementary Figure S2) and found that MH02ZBF003, MH06ZBF001, MH22ZBF001, MH10ZBF001 and MH20ZBF002 showed relatively high *In* values (>0.1).

### 3.4. Sequencing Results of the Developed Multiplex System Using the NGS Platform

Depth of coverage (DoC) and ACR were used to evaluate the sequencing results of the developed multiplex system (Supplementary Table S3). The mean DoC values ranged from 116 to 23,495. The rs1382755 and rs33911727 loci at the MH04ZBF001 locus showed low DoC values. For ACR, they ranged from 0.4573 to 0.9606. Most loci in these 29 loci showed relatively high ACR values, indicating relatively good intra-locus balances.

We also estimated Q30 of sequencing data for each individual. We found that they were greater than 90%, implying high accuracy. Some same individuals were analyzed by the developed system twice, and identical results of these loci were observed for the same individuals. Therefore, the developed system showed good performance and high genotyping accuracy.

### 3.5. Genetic Distributions and Forensic Parameters of the 29 Loci in Kazak and Mongolian Groups

HWE test results (*p*-values) of the 29 loci in Kazak and Mongolian groups are given in Table 2 and Table 3. After applying Bonferroni correction (*p* = 0.05/29 = 0.0017), the MH01ZBF002 locus deviated from HWE in the Kazak group, and the MH01ZBF002 and MH08ZBF002 loci deviated from HWE in the Mongolian group. LD analyses of pairwise loci in Kazak and Mongolian groups are given in Supplementary Tables S4 and S5. For the Kazak group, all pairwise loci conformed to linkage equilibrium after Bonferroni correction (*p* = 0.05/406 = 0.00012). However, one pair (MH06ZBF002 and MH07ZBF002) deviated from linkage equilibrium in the Mongolian group.

**Figure 5:**
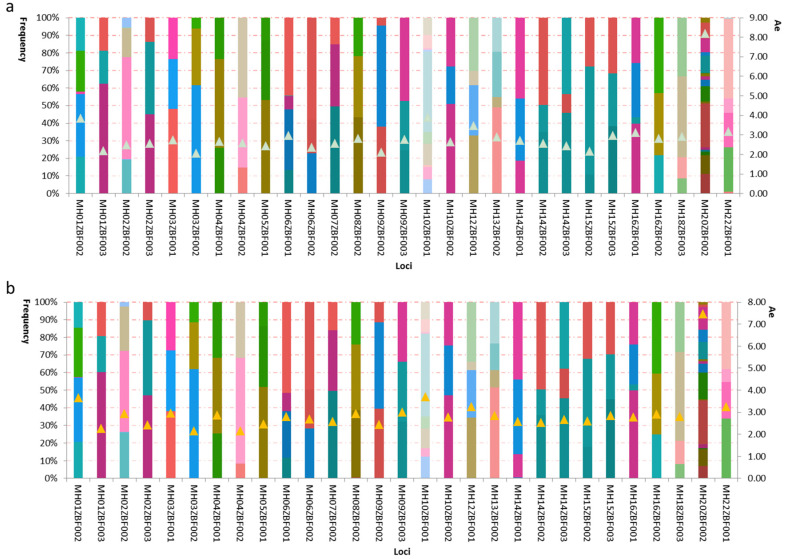
Haplotypic frequencies and the effective number of alleles at the 29 loci in (**a**) Kazak and (**b**) Mongolian groups. Stacked histogram indicated haplotypic frequencies of the 29 loci; the triangles in stacked histograms indicated the effective number of alleles at the 29 loci.

We plotted the stacked histograms of haplotypic frequencies and Ae values of the 29 loci in the Kazak and Mongolian groups (Figure 5). For the Kazak group, 3 to 18 alleles at the 29 loci could be observed, and their frequencies ranged from 0.0045 to 0.6250; Ae values of the 29 loci distributed from 2.05 at the MH03ZBF002 locus to 8.19 at MH20ZBF002 locus (Figure 5a). For the Mongolian group, a total of 116 alleles (3–15 alleles at each locus) were observed at the 29 loci with allelic frequencies ranging from 0.0047 to 0.6179; the smallest Ae (2.14) was at the MH03ZBF002 locus, while the largest Ae (7.45) was at the MH20ZBF002 locus (Figure 5b).

**Figure 6:**
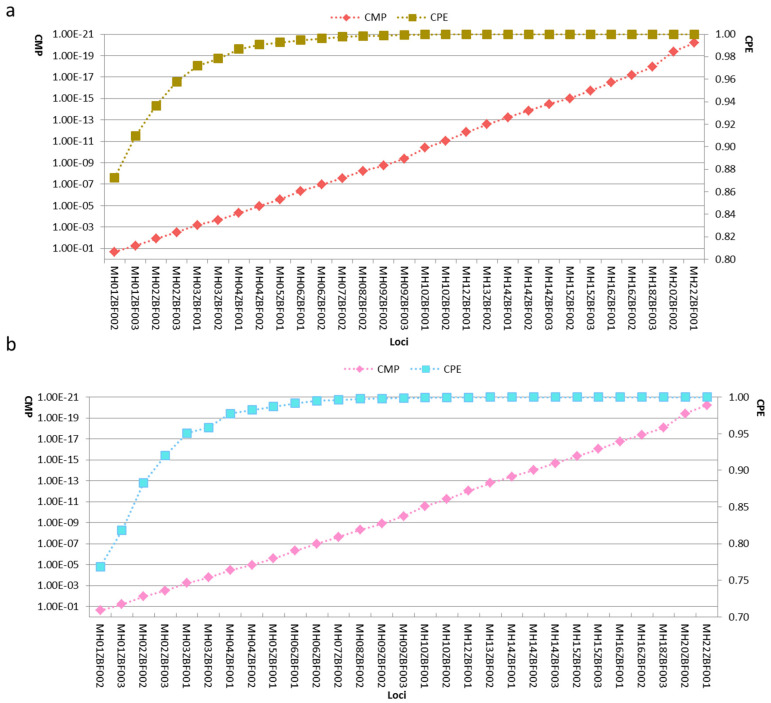
Cumulative match probability and probability of exclusion values of the 29 loci in (**a**) Kazak and (**b**) Mongolian groups.

The forensic parameters of the selected 29 loci in Kazak and Mongolian groups are presented in Table 2 and Table 3. The mean Ho, He, PIC, DP, MP and PE values of the 29 loci in the Kazak group were 0.6502, 0.6367, 0.5677, 0.7862, 0.2138 and 0.3689, respectively; they were 0.6490, 0.6439, 0.5743, 0.7897, 0.2103 and 0.3670 in the Mongolian group. There were four loci with PIC values <0.5 in both groups. Next, we calculated the CMP and CPE of the 29 loci, as shown in Figure 6. The results revealed that the CMP values of the 29 loci were less than 1.00E-20 and CPE values were close to 1 in both groups.

## 4. Discussion

STRs are the gold standard markers that are widely used in forensic DNA laboratories. The relatively larger amplicon size, stutter peak and high mutation rate exert the adverse influences on STR analysis. Microhaplotypes and compound markers are novel genetic markers that possess some advantageous forensic application features compared with STRs. Previous studies have constructed some panels of these novel genetic markers for different forensic research purposes [41,42]. In this study, we selected 29 novel loci including 22 microhaplotypes and seven compound markers (InDel-SNP) for forensic human identification and paternity testing in East Asian populations. We investigated genetic polymorphisms and forensic statistical parameters of the 29 loci in Kazak and Mongolian groups in China, and the results revealed that these loci showed relatively high polymorphisms in both groups.

The 29 loci presented in this study are distributed on 18 autosomal chromosomes. The physical distances between the 29 loci and the commonly used CODIS system on the same chromosomes were 10 Mb apart, implying that they were less likely to be in genetic linkage. Accordingly, these 29 loci could be used to forensic application along with these STRs.

Loci with high heterozygosity and relatively balanced allelic frequencies in populations could be viewed as valuable markers for forensic human identifications [4]. The PIC is an index that measures whether a marker is informative [43]. Ae is an indicator revealing loci usefulness in resolving DNA mixtures: the higher the Ae, the better the power of a locus to detect the mixture [32]. We assessed the He, Ae, and PIC values of 29 loci in five intercontinental populations (Figure 2 and Supplementary Figure S1). Not surprisingly, all loci demonstrated high He (>0.5) and PIC values (>0.5) in the East Asian population. There were one, two, five, and five loci with He values less than 0.5 in American, South Asian, African and European populations, respectively, and 9, 10, 11 and 13 loci with PIC values less than 0.5 in American, African, South Asian and European populations, respectively. Even so, all PIC values were larger than 0.25, suggesting that they were reasonably informative in these populations. The Ae values of the 29 loci in different continental populations revealed that most loci were relatively high (>2), especially for the East Asian population (Supplementary Figure S1). Therefore, the selected 29 loci could be used as informative markers for mixture deconvolution. The high CDP values of the 29 loci suggested that the panel could be regarded as a useful tool for human identifications in these populations. Moreover, the relatively high CPE values (>0.9999) implied that the panel was also appropriate for paternity analysis.

Population genetic analyses among five continental populations were conducted based on the 29 loci. According to PCA results, most East Asian and African individuals could be differentiated from other individuals at the first three PCs (Figure 3). Moreover, four continental populations (including African, European, South Asian and East Asian) displayed distinct genetic component distributions in the STRUCTURE analysis. Therefore, we inferred that some of the 29 loci may show large genetic variations among these populations, which led to population distribution patterns in PCA and STRUCTURE. *In* is generally considered as a parameter to evaluate genetic variations of the locus in different populations [44]. The *In* values of the 29 loci among these continental populations (Supplementary Figure S2) revealed that five loci had relatively high *In* values (>0.1), suggesting that they could be used as informative markers for ancestry inference of these continental populations.

Using the system presented in this study, most loci showed high DoC values. However, the rs1382755 and rs33911727 loci at MH04ZBF001 locus had low DoC values, implying low performance of the region during multiplex PCR and sequencing. We also observed that most loci showed relatively high ACR values (>0.66), indicating that they may be useful to analyze mixed sample. Next, we investigated genetic distributions of the 29 loci in Kazak and Mongolian groups. The results revealed that all loci had at least three allele variations in both groups, and MH20ZBF002 locus had the most alleles. The average Ae values of the 29 loci were 2.92 and 2.93 in the Kazak and Mongolian groups, respectively. According to the previous research published by Kidd et al. the cumulative probability of resolving a mixture is 0.9471 if there are five loci with Ae values of 3.00 [32]. In this study, there were five and six loci with Ae values greater than three in the Mongolian and Kazak groups, respectively, indicating the cumulative probability of the 29 loci to detect a mixture of two unrelated individuals theoretically was above 0.9471. We did not test the capability of these loci to resolve the mixture, which should be evaluated in future analyses. The CMP and CPE values of the 29 loci in the Kazak and Mongolian groups are shown in Figure 6. Compared to the results for 35 InDels and 30 InDels in the Kazak [24,45] and Mongolian groups [46,47], we found that the 29 loci had higher CDP and CPE values (Supplementary Table S6), suggesting that the panel could be employed for human identification and paternity analyses in the two groups.

## 5. Conclusions

We selected 29 novel loci including 22 microhaplotypes and seven compound markers for forensic application in the East Asian populations. We found that most of these 29 loci were relatively high polymorphisms in different continental populations. Moreover, five loci showed relatively high *In* values and could be used for ancestry inferences of these continental populations. Further evaluations of the 29 loci in Kazak and Mongolian groups yielded a similar conclusion: the 29 loci could be a valuable tool for human identification and paternity testing. The power of the 29 loci to detect the mixture needs to be validated.

## Figures and Tables

**Table 1:** Cumulative discrimination power, match probability and probability of exclusion values of 29 microhaplotypes and compound markers in five continental populations.

Continents	CDP	CMP	CPE
African	0.99999999999999999990425	9.5749 × 10^−20^	0.999982
American	0.9999999999999999999930322	6.9679 × 10^−21^	0.999983
European	0.99999999999999999982977	1.7023 × 10^−19^	0.999954
East Asian	0.99999999999999999999968073	3.1928 × 10^−22^	0.999998
South Asian	0.9999999999999999998964	1.036 × 10^−19^	0.999975

Note: CDP, cumulative discrimination power; CMP, cumulative match probability; CPE, cumulative probability of exclusion.

**Table 2:** Forensic parameters of 29 microhaplotypes and compound markers in Kazak group.

Loci	He	Ho	PIC	MP	DP	PE	*p*
MH01ZBF002	0.7425	0.9375	0.6935	0.2044	0.7956	0.8725	0.0000
MH01ZBF003	0.5415	0.5982	0.4817	0.2761	0.7239	0.2887	0.5120
MH02ZBF002	0.5988	0.6071	0.5486	0.2167	0.7833	0.2995	0.6480
MH02ZBF003	0.6116	0.6339	0.5260	0.2588	0.7412	0.3336	0.0380
MH03ZBF001	0.6353	0.6339	0.5608	0.2068	0.7932	0.3336	1.0000
MH03ZBF002	0.5155	0.5446	0.4310	0.3310	0.6690	0.2296	0.4680
MH04ZBF001	0.6253	0.6786	0.5524	0.2251	0.7749	0.3959	0.6220
MH04ZBF002	0.6158	0.5982	0.5318	0.2250	0.7750	0.2887	0.7210
MH05ZBF001	0.5885	0.5357	0.5076	0.2414	0.7586	0.2207	0.3690
MH06ZBF001	0.6658	0.5804	0.6015	0.1674	0.8326	0.2680	0.1970
MH06ZBF002	0.5771	0.6071	0.5109	0.2481	0.7519	0.2995	0.6970
MH07ZBF002	0.6097	0.6518	0.5289	0.2463	0.7537	0.3577	0.8450
MH08ZBF002	0.6463	0.6161	0.5683	0.2127	0.7873	0.3106	0.0670
MH09ZBF002	0.5247	0.5357	0.4250	0.3276	0.6724	0.2207	0.9010
MH09ZBF003	0.6395	0.7143	0.5647	0.2242	0.7758	0.4507	0.3740
MH10ZBF001	0.7457	0.7589	0.7211	0.0969	0.9031	0.5252	0.0140
MH10ZBF002	0.6212	0.6161	0.5480	0.2229	0.7771	0.3106	0.3440
MH12ZBF001	0.7138	0.7589	0.6540	0.1583	0.8417	0.5252	0.0400
MH13ZBF002	0.6545	0.6250	0.5942	0.1830	0.8170	0.3220	0.4470
MH14ZBF001	0.6318	0.6518	0.5528	0.2160	0.7840	0.3577	0.5690
MH14ZBF002	0.6115	0.6161	0.5313	0.2403	0.7597	0.3106	0.6730
MH14ZBF003	0.5922	0.5446	0.5011	0.2417	0.7583	0.2296	0.3770
MH15ZBF002	0.5348	0.5804	0.4637	0.2953	0.7047	0.2680	0.4940
MH15ZBF003	0.6656	0.6696	0.5887	0.1923	0.8077	0.3829	0.6800
MH16ZBF001	0.6820	0.6964	0.6143	0.1711	0.8289	0.4228	0.8670
MH16ZBF002	0.6470	0.6429	0.5689	0.2038	0.7962	0.3455	0.9240
MH18ZBF003	0.6577	0.6607	0.5931	0.1786	0.8214	0.3701	0.7510
MH20ZBF002	0.8818	0.8839	0.8674	0.0362	0.9638	0.7627	0.0080
MH22ZBF001	0.6858	0.6786	0.6308	0.1518	0.8482	0.3959	0.8050

Note: He—expected heterozygosity; Ho—observed heterozygosity; PIC—polymorphism information content; MP—match probability; DP—discrimination power; PE—probability of exclusion; *p*—*p*-value for Hardy–Weinberg equilibrium test.

**Table 3:** Forensic parameters of 29 microhaplotypes and compound markers in Mongolian group.

Loci	He	Ho	PIC	MP	DP	PE	*p*
MH01ZBF002	0.7296	0.8868	0.6769	0.2415	0.7585	0.7685	0.0000
MH01ZBF003	0.5596	0.5283	0.4966	0.2560	0.7440	0.2135	0.5600
MH02ZBF002	0.6610	0.6509	0.5934	0.1808	0.8192	0.3565	0.8810
MH02ZBF003	0.5893	0.6226	0.4976	0.2768	0.7232	0.3189	0.5320
MH03ZBF001	0.6637	0.6698	0.5864	0.1928	0.8072	0.3831	0.9040
MH03ZBF002	0.5356	0.4623	0.4662	0.2789	0.7211	0.1566	0.2500
MH04ZBF001	0.6541	0.7170	0.5773	0.2140	0.7860	0.4550	0.5280
MH04ZBF002	0.5366	0.5377	0.4557	0.2992	0.7008	0.2227	0.9450
MH05ZBF001	0.5963	0.5755	0.5151	0.2391	0.7609	0.2625	0.9170
MH06ZBF001	0.6442	0.6415	0.5875	0.1817	0.8183	0.3437	0.4410
MH06ZBF002	0.6284	0.6792	0.5542	0.2250	0.7750	0.3969	0.7500
MH07ZBF002	0.6133	0.5849	0.5335	0.2225	0.7775	0.2732	0.8860
MH08ZBF002	0.6604	0.6415	0.5837	0.2164	0.7836	0.3437	0.0000
MH09ZBF002	0.5923	0.5660	0.5038	0.2583	0.7417	0.2521	0.4110
MH09ZBF003	0.6696	0.6321	0.5924	0.1857	0.8143	0.3312	0.2430
MH10ZBF001	0.7316	0.7075	0.7043	0.1150	0.8850	0.4400	0.0100
MH10ZBF002	0.6403	0.6698	0.5652	0.2147	0.7853	0.3831	0.6320
MH12ZBF001	0.6948	0.6698	0.6290	0.1563	0.8437	0.3831	0.5500
MH13ZBF002	0.6505	0.6132	0.5967	0.1780	0.8220	0.3070	0.3370
MH14ZBF001	0.6128	0.6604	0.5275	0.2496	0.7504	0.3697	0.8110
MH14ZBF002	0.6077	0.5849	0.5258	0.2433	0.7567	0.2732	0.2290
MH14ZBF003	0.6267	0.5943	0.5453	0.2090	0.7910	0.2841	0.5610
MH15ZBF002	0.6179	0.6226	0.5409	0.2223	0.7777	0.3189	0.4660
MH15ZBF003	0.6491	0.5660	0.5730	0.1924	0.8076	0.2521	0.0560
MH16ZBF001	0.6428	0.6698	0.5785	0.2010	0.7990	0.3831	0.5270
MH16ZBF002	0.6575	0.7358	0.5800	0.2218	0.7782	0.4859	0.1210
MH18ZBF003	0.6443	0.7453	0.5843	0.2205	0.7795	0.5017	0.0370
MH20ZBF002	0.8699	0.8585	0.8527	0.0418	0.9582	0.7117	0.0080
MH22ZBF001	0.6939	0.7264	0.6314	0.1630	0.8370	0.4703	0.1710

Note: He—expected heterozygosity; Ho—observed heterozygosity; PIC—polymorphism information content; MP—match probability; DP—discrimination power; PE—probability of exclusion; *p*—*p*-value for Hardy–Weinberg equilibrium test.

## Supplementary Materials

The following are available online at https://www.mdpi.com/2073-4425/11/9/1027/s1. Supplementary Figure S1. Heatmap of effective number of alleles at the 29 loci in five continental populations. Supplementary Figure S2. Informativeness (*In*) values of the 29 loci in five continental populations. Supplementary Table S1. Primer information of 69 SNP/InDel loci at 29 microhaplotypes and compound markers. Supplementary Table S2. General information of 29 microhaplotypes and compound markers. Supplementary Table S3. Depth of coverage and allele coverage ratio distributions of 69 SNP/InDel loci at 29 microhaplotypes and compound markers. Supplementary Table S4. Linkage disequilibrium analyses (*p*-values) of pairwise loci in Kazak group. Supplementary Table S5. Linkage disequilibrium analyses (*p*-values) of pairwise loci in Mongolian group. Supplementary Table S6. Forensic efficiency comparisons of the 29 microhaplotypes and compound markers in this study and other published panels in Kazak and Mongolian groups.
