# Supplementary figures and images for: A Highly Polymorphic Panel Consisting of Microhaplotypes and Compound Markers with the NGS and Its Forensic Efficiency Evaluations in Chinese Two Groups
Source: Genes (Basel). 2020 Sep 1;11(9):1027. doi: 10.3390/genes11091027 (PMC7565596; doi:10.3390/genes11091027)

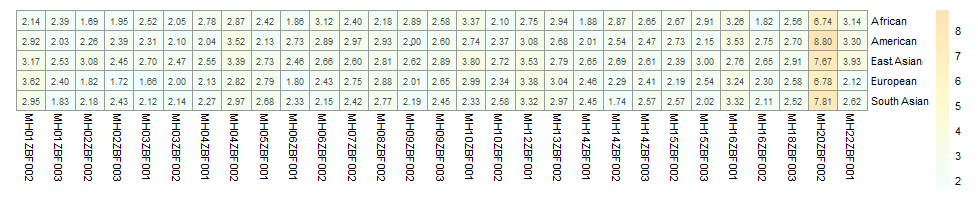

Supplement: Supplementary file 1 [file genes-11-01027-s001.zip › Supplementary Materials/Supplementary Figure 1.png]

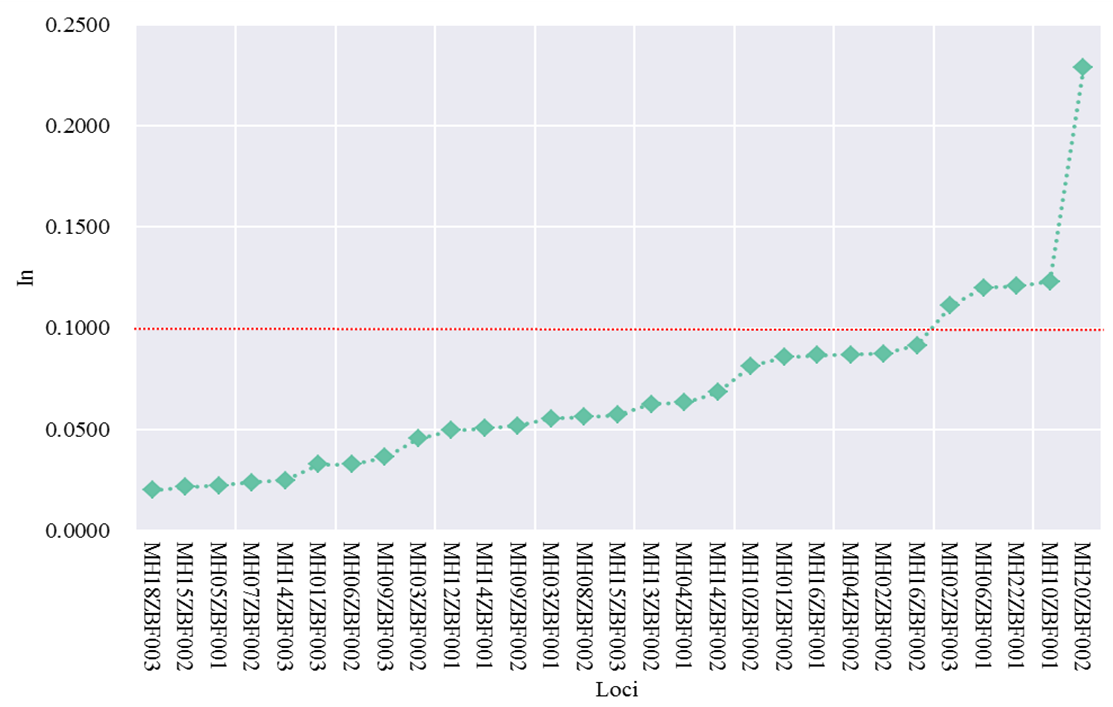

Supplement: Supplementary file 1 [file genes-11-01027-s001.zip › Supplementary Materials/Supplementary Figure 2.png]
